# How has the COVID‐19 pandemic affected eczema self‐management and help seeking? A qualitative interview study with young people and parents/carers of children with eczema

**DOI:** 10.1002/ski2.59

**Published:** 2021-06-23

**Authors:** M. Steele, L. Howells, M. Santer, K. Sivyer, S. Lawton, A. Roberts, E. Teasdale, I. Muller, K. Greenwell

**Affiliations:** ^1^ Primary Care, Population Sciences and Medical Education Faculty of Medicine University of Southampton Southampton UK; ^2^ Centre for Clinical and Community Applications of Health Psychology School of Psychology Faculty of Environmental and Life Sciences University of Southampton Southampton UK; ^3^ Centre of Evidence Based Dermatology School of Medicine University of Nottingham Nottingham UK; ^4^ Department of Psychology Faculty of Science and Health University of Portsmouth Portsmouth UK; ^5^ Rotherham NHS Foundation Trust Rotherham UK; ^6^ Nottingham Support Group for Carers of Children with Eczema Nottingham UK

## Abstract

**Background:**

Eczema can have a considerable impact on quality of life. Treatments can improve this, but management is complex. Barriers to eczema self‐management may be impacted upon by environmental context, such as the COVID‐19 pandemic.

**Objectives:**

To explore experiences of eczema, self‐management, and accessing healthcare and advice during the COVID‐19 pandemic among young people with eczema and parents/carers of children with eczema.

**Methods:**

Qualitative semi‐structured interviews were carried out with 36 participants recruited from general practices as part of randomised controlled trials of online eczema resources.

**Results:**

*Changes to everyday life—*Periods of staying at home due to the pandemic alter the burden of eczema, with reports of an improved routine and application of topical treatments for many, but difficulties with handwashing for others. Parents/carers reported improved eczema control due to closures of educational settings. Young people reported higher stress that may have triggered eczema flare‐ups. *Changes to access to advice and treatment—*There was a reluctance to seek medical appointments in a non‐emergency situation. Participants reported a lack of trust in the outcome of telephone consultations because health professionals were unable to see or feel the skin. Delays or difficulties when obtaining appointments and treatments caused frustration. Access to an online eczema resource was reported to have extra value in the context of the pandemic.

**Conclusion:**

Changes to lifestyle and access to healthcare during the pandemic have affected eczema and self‐management. Healthcare settings may want to consider providing extra reassurance around remote consultations.

## Graphical abstract

This interview study explored experiences of eczema, self‐management, and accessing healthcare and advice during the covid‐19 pandemic among young people with eczema and parents/carers of children with eczema. Both groups reported how changes to their lifestyle and access to healthcare during the pandemic affected eczema and self‐management. Healthcare services should provide extra reassurance around remote consultations and advice on how to deal with things during the pandemic that might trigger eczema, such as increased handwashing.

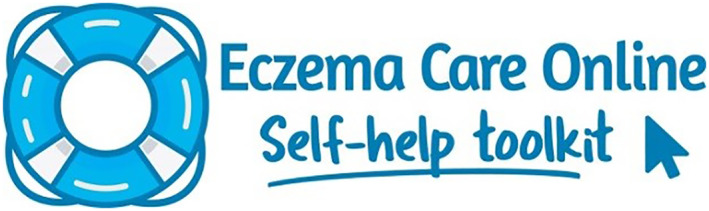

1

**What is already known about this topic?**

- Effective eczema self‐management involves regular application of topical treatments and avoidance of triggers.
- People with eczema and their carers often find eczema difficult to manage, and treatments can be a challenge to fit into everyday life.
- Some of the barriers to eczema self‐management, such as the time‐consuming nature of treatments or difficulties avoiding triggers, are affected by environmental context.

**What does this study add?**

- Pandemic‐related restrictions meant some people said they had more time for eczema treatments and less exposure to triggers outside the home, highlighting the context‐specific nature of treatment burden.
- Increased handwashing triggered hand eczema and some young people felt that stress caused by the pandemic worsened their eczema.
- Remote consultations were viewed as less satisfactory than face‐to‐face consultations, with a concern that health professionals can't fully assess and advise on treatments.

**What are the clinical implications of this work?**

- There may be value in providing advice to minimise the impact that changes during the pandemic may have on eczema, for instance around handwashing.
- The emotional impact of the pandemic needs to be acknowledged and support provided with stress management.
- Reassurance before and/or during remote consultations could boost patient or carer confidence in consultation outcomes.
- Online eczema resources can provide reassurance when people are reluctant to seek healthcare professionals support.

## 2 INTRODUCTION

Eczema is a skin condition that can considerably affect quality of life for the person with eczema and their family^1^ but effective treatments and self‐management of eczema can improve this. Eczema self‐management can be complex. Key components of self‐management are the avoidance of triggers, such as soap, and application of topical treatments, including regular use of emollients and application of topical corticosteroids for flare‐ups.^2^ The National Institute for Health and Care Excellence guidelines suggest that the underuse of treatments is the main cause of treatment failures. Previous research has identified barriers to self‐management, including limited knowledge of eczema, concerns about eczema treatments and treatments being time‐consuming.^3^, ^4^, ^5^, ^6^

Recent evidence suggests the COVID‐19 pandemic has had an impact on the mental health and emotional wellbeing of young people,^7^, ^8^ and there have been calls to investigate the implications of this for chronic skin conditions.^9^ We interviewed parents/carers of children with eczema and young people with eczema during the pandemic, and explored the impact of the pandemic on their experience of eczema, eczema management and seeking health care for eczema.

The current interview study was embedded in randomised controlled trials of the Eczema Care Online (ECO) digital self‐management interventions, which began recruitment in November 2019.^10^ Qualitative data collection started in March 2020, coinciding with the first UK lockdown due to the COVID‐19 pandemic (see Box 1) that changed the living circumstances of many participants. Therefore, questions were added to the interviews, providing a unique opportunity to examine how the pandemic affected participants, the management of eczema and the way in which they accessed healthcare during the pandemic.

## 3 PATIENTS AND METHODS

### 3.1 Study design and data collection

The ECO research programme developed and trialled two online behavioural interventions using theory‐, evidence‐ and person‐based approaches.^10^, ^11^, ^12^ One is for parents/carers of children with eczema and the second is for young people with eczema. The interventions aimed to reduce eczema severity by supporting self‐management, including use of emollients and topical corticosteroids, management of triggers, emotional/stress management and reduced scratching.

The study design was a qualitative interview study and was part of a mixed‐methods process evaluation embedded in the ECO randomised controlled trials.^10^ Over 600 ECO trial participants were recruited via mail‐out from 98 general practices across Wessex, West of England, East Midlands, and Thames Valley and South Midlands. Eligible participants included young people aged 13 to 25 with eczema and parent/carers of children with eczema aged 0 to 12 years. Young people and children had to have a confirmed diagnosis of eczema, have obtained a prescription for eczema treatment in the past 12 months, and have a POEM score (measuring eczema severity)^13^ of greater than five. Participants consented online, and parental consent was sought for young people aged under 16.

Interviewees were purposively sampled from trial participants who had consented to be approached for an interview. Sampling ensured a range of ages, ethnicity, socioeconomic status, eczema severity, intervention usage, trial group (intervention and usual care) and a balance of genders. Process evaluation interview topics included views and experiences of the ECO intervention, perceived changes following the intervention, facilitators and barriers to use, and views on implementation of the intervention. Additional questions were added in March 2020 (Box 2) to evaluate how the COVID‐19 pandemic affected participants' experiences of eczema and its management, and the use of the ECO interventions. A patient and public involvement (PPI) representative (AR) reviewed and provided feedback on the interview topic guide.

Data collection took place from 24 March 2020 to 2 February 2021. Interviews were spread out across this time period to capture perspectives from different stages of the ongoing pandemic. Phone interviews were carried out due to the pandemic social distancing rules (see Box 1), were audio‐recorded and transcribed verbatim. The interviewing team (M.St, L.H, K.G, K.S) were female post‐doctorate researchers and three were involved in the development of the ECO interventions. Interviewers wrote detailed summaries after each interview and met weekly to discuss these, refining the interview topic guides and sampling strategy where necessary. Interviews were carried out until meaning saturation of the process evaluation data was reached, which was defined as being able to fully understand participants' diverse intervention experiences.^14^ The study was approved by the South Central Oxford A Research Ethics Committee (19/SC/0351).

### 3.2 Data analysis

Data were analysed by following the six stages of Braun and Clarke’s thematic analysis.^15^ M.St familiarised herself with the transcripts and extracted all data relating to COVID‐19. NVivo (version 12) was used to manage the data. Using an inductive approach, line‐by‐line coding was carried out and codes organised into broader themes. Codes and themes were presented and discussed with a team consisting of a general practitioner, health researchers, and psychologists. A PPI representative (AR) reviewed the study findings and provided feedback on interpretations. Such a collaborative approach aimed to develop a richer more nuanced understanding of the data.^16^

## 4 RESULTS

Twenty young people and 20 parents/carers took part in the ECO process evaluation interviews. Thirty‐four of these (15 young people, 19 parents/carers) included data on the COVID‐19 pandemic (Table 1). The remaining six interviews were carried out before the questions relating to COVID‐19 were added to the topic guide and did not include any mention of the pandemic or lockdown, so were not included in the analysis. The analysis team felt that code saturation^14^ was reached, as no additional codes were generated when analysing the remaining transcripts. However, it was agreed that additional interviews were needed to fully understand the nuances of the pandemic experiences for the groups (i.e., meaning saturation was not reached for this group^14^).

**BOX 1 Outline of the effects of the COVID‐19 pandemic on the United Kingdom**

On 26 March 2020, the UK government enforced a lockdown due to the COVID‐19 pandemic. This resulted in a temporary, but significant, change in circumstances for many of the population including:

- the closure of educational settings,
- a move to home or remote working, and
- being placed on a furlough scheme where many people were not able to work for several months.

The public were asked to take measures to prevent the spread of the virus such as:

- wearing facemasks in public indoor places,
- social distancing, and
- increased handwashing.

Healthcare settings had to change the way they delivered services, with face‐to‐face appointments being replaced by remote consultations (online or via telephone) whenever possible. Over the course of the next year, COVID‐19 restrictions were enforced, with rules being relaxed and tightened, dependant on the spread of the pandemic, and was ongoing at the time of writing this paper.

**BOX 2 Subset of interview questions relevant to the COVID‐19 pandemic**

Q1. We're interested in how people are finding looking after their eczema during the coronavirus lockdown. Can you tell me about how your [child's] eczema has been during this time?
  - *Prompts*: change in circumstances? extra handwashing? access to creams/medical advice? change in stress?
  - [If changed] What do you think led to your [child's] eczema getting better/worse?
Q2. Can you tell me about anything you are doing differently to look after your [child's] eczema?
Q3. We understand that everyone's experiences and home situations during lockdown are very different. What aspects of lockdown have made it more difficult for you to manage your [child's] eczema?
Q4. What aspects of lockdown have made it easier for you to manage your [child's] eczema?
Q5. How have you found using the ECO website during lockdown?
  - Can you tell me about any information or advice that was difficult for you to follow during lockdown?

**TABLE 1:** Participant characteristics

	Young people	Parents/carers
Participant age		
Mean age (years)	18.33	38.53
Age range (years)	13–25	25–62
Child age		
Mean age (years)	N/A	4.72
Age range (years)	N/A	0–12
Participant gender		
Female *N* (%)	7 (46.67)	15 (78.95)
Child gender		
Female *N* (%)	N/A	9 (47.37)
Ethnic background (self‐defined)		
White British *N* (%)	10 (66.67)	14 (73.68)
Indian *N* (%)	3 (20.00)	1 (5.26)
Chinese *N* (%)	0	2 (10.53)
African *N* (%)	1 (6.67)	1 (5.26)
White & Asian *N* (%)	1 (6.67)	0
White & Black Caribbean *N* (%)	0	1 (5.26)
Trial group		
Intervention *N* (%)	14 (93.33)	17 (89.47)
Usual care *N* (%)	1 (6.67)	2 (10.53)
Eczema severity (defined by POEM)^a^		
Mild *N* (%)	2 (13.33)	4 (21.05)
Moderate *N* (%)	6 (40)	10 (52.63)
Severe *N* (%)	7 (46.67)	5 (26.32)
Socioeconomic status (index of multiple deprivation score^b^)		
Mean	7.14	6.63
Range	2–10	1–10

^a^ Mild eczema defined as POEM 6–7; Moderate eczema POEM 8–16; Severe eczema POEM 17–28. Respondents with very mild eczema (a POEM score of 5 or lower) were excluded from the research.

^b^ 10 is the highest socioeconomic status.

**FIGURE 1:**
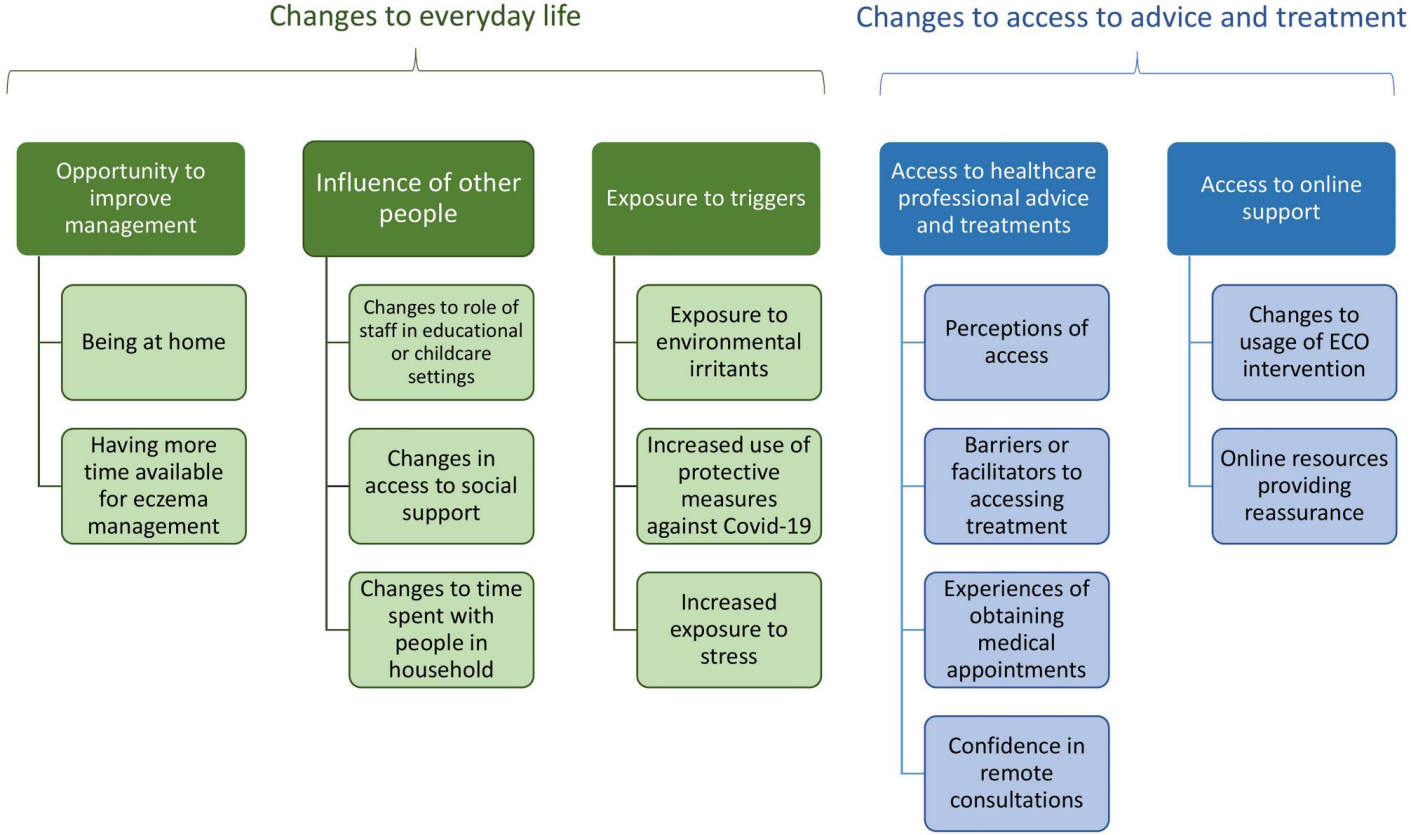
Key themes and findings

Under the broad categories of ‘changes to everyday life’ and ‘changes to access to advice and treatment’, five key themes were identified (Figure 1).

### 4.1 Changes to everyday life

Generally, both young people and parents/carers reported that spending increased time at home due to the pandemic lockdown restrictions improved their eczema management routine and most reported increased use of emollients. For young people, not leaving the house as they normally would in the mornings or evenings had facilitated their increased use of emollients.

I've had more time in the mornings to, you know, spend time, like, putting my moisturiser on, rather than kind of waking up late, skipping that bit and then just running for the bus. YP12 (25‐year‐old, moderate eczema)

I'm not going out as much [in the evenings], so I'm a lot more likely to remember to put it [emollients] on at night. YP20 (20‐year‐old, severe eczema)

Participants also said that the ease of having treatments and bathing facilities close to hand when at home also made eczema care more straightforward.

[It was] much easier, as well, if she [my daughter] was irritated, to have medication during the day or go and put extra cream on during the day or go and have a quick shower or whatever. So, in some ways, there was more flexibility, looking after it [eczema] at home, than if she was out and about. PC13 (parent/carer of a 10‐year‐old, severe eczema)

Parents/carers said that having their children at home from school/nursery and being able to take full control of their eczema care, rather than relying on the school/nursery staff or the child to remember, was helpful.

We've had the odd day where obviously a teacher hadn't been on top of it [eczema] and, you know, [name of son] would come back, having scratched himself loads during that day, for whatever reason. So we haven't had that [during the pandemic]. PC08 (parent/carer of child age 2, moderate eczema)

The nurseries closed [during the pandemic] and he [her son] was with me all the time. So I was there to put it on him, put the emollients on him, a lot more than what maybe I would have done if he'd been at nursery. PC19 (parent/carer of child age 2, moderate eczema)

Due to the pandemic, participants were unable to have close contact with people outside of their households for extended time periods. Some described that the loss of physical contact with their support network, such as grandparents who assisted with childcare, made it more difficult to cope with eczema symptoms such as sleep disturbance.

So when I've have, sort of, multiple restless nights [due to child not sleeping because of itching], my mum would then say, oh, bring her round to mine for the night and you get some sleep. So…we can't really have that kind of outside support and I think that's hard. PC20 (parent/carer of child age 2, moderate eczema)

Often, entire households were at home together more than they would have before the pandemic. Participants described how having other people present served as a reminder to apply creams and improved their awareness of habits such as scratching, which can also trigger eczema flare‐ups.

It's made me more aware of, when I'm, because now I'm [at home with my] family and, you know, they make me aware as well that, you know, you shouldn't do that or don't itch. YP07 (24‐year‐old, severe eczema)

There was another person in the house to actually say…have we done [son's name] cream? And to remind me. PC06 (parent/carer of child under 1, severe eczema)

When participants reported eczema improvements or flare‐ups, they tended to speculate on what may have caused these. In addition to the increased opportunity to apply topical treatments afforded by being at home more, improvements were often attributed to reduced exposure to triggers outside of the home. Soaps were also commonly mentioned as examples of triggers.

[My son has] not been going to nursery or anything and using different soaps and that type of thing. So it's [eczema] been quite well‐controlled through lockdown. PC10 (parent/carer of a 2‐year‐old, moderate eczema)

Conversely, some participants attributed eczema flare‐ups to irritants in their home environment including exposure to central heating, increased washing up, and a lack of vitamin D due to being indoors. Some participants also reported increased scratching due to boredom.

So obviously at home, she's [her daughter] bored; she can get easily frustrated. So I suppose, in that sense, yes [her eczema has been affected], because she's not out, having fun and being distracted, whereas at home, she can just be at home, bored and then when she's bored and she can just start itching. PC20 (parent/carer of a 2‐year‐old, moderate eczema)

Protective measures used to prevent the spread of COVID‐19 were perceived to have worsened eczema in both groups. One participant described skin irritation due to wearing protective masks at work. The most common trigger mentioned was increased handwashing with soap or hand gel. Parents/carers reported that their children's hand eczema improved in the periods of lockdown when children were at home and, therefore, washed their hands less.

Using hand sanitiser when you go out, as well, not having handwashing facilities, hand sanitiser literally will strip my eczema; it makes my skin so bad. YP11 (23‐year‐old, moderate eczema)

Handwashing has made a massive [difference] which, again, is worse if she's [her daughter] out and about, because they don't have to wash, well, I don't know [if] they wash their hands as often home as they do at school. PC13 (parent/carer of child age 10, severe eczema)

Young people (mainly those with severe eczema) often referred to stress as a trigger for eczema flare‐ups, which for most was caused by general worry and uncertainty regarding the pandemic. Young people also reported worries about the health of family members or feeling stressed about situations at school/university caused by the pandemic.

So I [have been] really quite stressed [about an event being cancelled due to the pandemic] and crying and just being angry and then that stress now, over emotional stuff, makes my eczema and my scratching and my allergic stuff 10 times worse. YP03 (15‐year‐old, severe eczema)

Obviously I [was] really stressed…because we didn't really know what was happening [with university] or anything like that. YP05 (19‐year‐old, severe eczema)

There was no mention of stress in children. When asked, parents/carers did not think that the pandemic had caused stress in their children.

### 4.2 Changes to access to eczema advice and treatments

A common perception among both groups was that GP appointments would be difficult to obtain during the pandemic or that they should not try to obtain a non‐emergency appointment. Some participants cited a lack of communication from their general practice as a reason for this perception. Participants reported feeling cut off from NHS services and taking extra measures to ensure that their skin, or their child's skin, did not get worse.

There wasn't really…much coming from the doctor's side of things as to, you can still ring us with this type of problem; we will do our best to sort of help you. I kind of felt like we were just, almost, like, abandoned and told just get on with it and see how it goes, sort of thing. PC06 (parent/carer of 11 month old, severe eczema)

I just keep running in out of her [her daughter] room, checking her, make sure she's not scratching, make sure she's not make it [eczema] worse, because now, if it does get that bad, I mean I'm sure I can take her to the doctor but that's in the back of your mind, I don't know if I can, can I? Can I just take her? It's not as easy as it was before. So you have to really make sure it doesn't get that bad. PC20 (parent/carer of a 2‐year‐old, moderate eczema)

Most participants who voiced doubts about being able to obtain medical appointments had not attempted or needed to do so. Only a few reported experiencing such difficulties.

So I phoned up the GP…this morning, and I was number 22 in the queue and I just can't…wait…number 22, it just takes forever and I've got, I couldn't wait. PC19 (parent/carer of a 2‐year‐old, moderate eczema)

Some parents/carers of very young children explained that their regular in‐person contact with the health visitor had been replaced by telephone calls.

No one has actually seen her [my daughter] in a very long time, physically, like in person. So…we are kind of very much on our own in terms of assessing how her eczema is and … other problems. PC18 (parent/carer of an 1‐year‐old, moderate eczema)

Participants in both groups expressed concerns about the telephone consultations that replaced face‐to‐face consultations during the pandemic, with many displaying a lack of confidence in the accuracy of prescriptions for treatments or the diagnosis of eczema‐related problems such as infections. They explained that the doctor being unable to touch or see the skin in person was the reason for this, despite the option to send photographs in.

Before [the pandemic], the GP can see and touch the skin; that may be more accurate, but after the lockdown, we only sent the picture [of their child's eczema to the GP] PC12 (parent/carer of a 4‐year‐old, severe eczema)

Well I guess it's like they [health professionals] can't feel it [eczema] themselves…I've taken her [her daughter] on numerous appointments about her skin and they sort of feel, like, examine it, feel it for themselves, have a good look, whereas this one, it's just a picture. There's lighting to take into consideration; it looks different on a picture. PC20 (parent/carer of a 2‐year‐old moderate eczema)

In particular, when the doctor prescribed the same treatment as they had previously been given, or a steroid that they deemed to be too mild, participants perceived this to be due to their inability to diagnose the problem accurately and perhaps the doctor being reluctant to prescribe a stronger treatment without seeing the skin in person.

I think it was harder for them to, it wasn't really harder, because they couldn't see the skin, they didn't know what [treatment] to accurately give us; they just gave us a refill of the ointment that I was using. YP04 (14‐year‐old, moderate eczema)

Because of COVID it's not easy [to] get to the GP or whatever; they've not really looked at it [eczema]. So they only prescribe the hydrocortisone cream. PC16 (parent/carer of an 1‐year‐old, moderate eczema)

Even when participants were prescribed treatments that cleared up the skin, they were still not confident in the accuracy of remote consultations.

Some participants reported that treatments and other products were harder to obtain. Participants reported shortages of eczema‐friendly hand cleaning products, long queues at pharmacies, and delays in treatments arriving at pharmacies. Some young people experienced delays because of registering with a different general practice due to relocating home from university.

Most participant reports of obtaining repeat prescriptions from their general practice were positive. This was usually due to automated or online systems. In contrast, other participants found that obtaining repeat prescriptions was difficult and expressed frustration at the process used by their general practice. Although no participants reported adverse effects from the delays in treatment, some had almost run out of some treatment due to these delays.

Ringing the doctors, it's just a nightmare because of COVID. But I don't know why they can't just let me have a repeat prescription and just pop into the pharmacy and get some more. PC19 (parent/carer of a 2‐year‐old, moderate eczema)

[We] haven't ever completely run out of the hydrocortisone, but we have had days when we been having to search around for any, almost, empty tubes, because we've been delayed with getting the prescription. PC17 (parent/carer of a 6‐year‐old, moderate eczema)

### 4.3 Accessing online support

There were varied experiences of accessing online support during lockdown. Some participants in the trial intervention group found that they were too busy to use the ECO intervention, and some who had more time at home thought that the pandemic had decreased their usage of the intervention due to worry or preoccupation.

I think I was just going out of my mind during lockdown and, in all honesty, the [ECO] website, it was at the back of my mind at the time. YP18 (14‐year‐old, severe eczema)

Some participants said they had more time to look at information about eczema online. A few parents of older children described using their child's increased time at home to involve the child in their own eczema care and using the ECO website.

I think I looked at it [ECO website] more in lockdown than I did when I'm at school. YP17 (13‐year‐old, moderate eczema)

Because he's [her son] been at home a lot in lockdown…we sat down and had a discussion about it [ECO website] and then I showed him the video the What Is Eczema video. PC03 (parent/carer of a 4‐year‐old, mild eczema)

Several participants described thinking of the ECO website as an alternative source of eczema advice and information to consulting with medical professionals. This was particularly useful and reassuring to those who were unsure about whether they would be able to obtain healthcare professional advice during the pandemic.

Perhaps if I'd have joined the study a little bit earlier and had the [ECO] website a bit earlier in lockdown, I might have used it a bit more, thinking obviously I can't…contact the healthcare professionals…because of the coronavirus, then maybe I would have used it a little bit more at the start. PC06 (parent/carer of 11 month old, severe eczema)

## 5 DISCUSSION

### 5.1 Summary of findings

Both parents/carers of children with eczema and young people with eczema noticed changes in eczema due to reduced exposure to irritants and triggers outside of the home during the pandemic and increased handwashing as a protective measure against COVID‐19. Young people noted how increased stress or worry caused directly or indirectly by the pandemic led to a worsening of eczema. Participants reported changes to seeking healthcare and advice relating to eczema during the pandemic. A reluctance to contact general practices for non‐emergency treatment and uncertainty about the availability of healthcare professional advice was reported, leading to feelings of worry about being ‘abandoned’ to manage the eczema alone in some cases. Remote consultations were commonly viewed as less satisfactory than face‐to‐face consultations, with a concern that if health professionals were unable to see and feel the skin they could not assess it properly, even when photographs were provided.

### 5.2 Findings in the context of existing research

The reported improvements to routine and increased use of treatments due to the removal of barriers to eczema self‐management during periods of staying at home highlight the hidden burden of self‐managing eczema and the importance of environmental factors.^17^, ^18^, ^19^ There is a need for high quality consistent information and advice about eczema.^18^, ^20^, ^21^ Eczema has a considerable psychosocial impact,^18^, ^22^ particularly amongst young people,^23^ and families of children with eczema often need to rely on others for support with eczema care.^22^, ^24^ All of these are affected by living through a pandemic, as formal healthcare can be harder to access, stresses can be increased and support networks are disrupted. Our findings relating to the added stress and worry for young people due to disruption to everyday life and to education are similar to themes found in other qualitative work exploring the experiences of young people during the pandemic.^7^, ^8^

During the pandemic, dermatology departments and primary care services were advised to carry out most appointments remotely,^25^, ^26^ although this is not welcome to all patient groups and that remote consultations include risks, both to the therapeutic relationship and also to the nature of the assessment.^25^ Younger people can be more likely to welcome remote assessment for its convenience,^27^ yet our findings suggest that, at least for some younger parents of children with eczema or young people with eczema, this is not the case.

### 5.3 Strengths and limitations

This study adds to the limited evidence‐base on the impact of global pandemic conditions on people's experiences of eczema and eczema management. Data were taken from a process evaluation study where exploring COVID‐19 experiences was not the primary aim, however it adds to a limited literature examining the impact of the pandemic on eczema patients and their carers. Participants were recruited from a trial sample who are likely to be highly motivated to engage with research and eczema treatments and may not be representative of the wider eczema population.^28^ However, the large trial sample size meant that we were able to sample participants with diverse demographics.

### 5.4 Implications for research, policy and practice

Barriers to accessing treatments can be mitigated by implementing accessible online or automated systems in general practices that would save time and effort for both patients and surgery staff. Further research into the reasons behind reluctance to contact healthcare professionals during the pandemic is needed. People may benefit from clear guidance and reassurances, where possible, on access to healthcare in a pandemic situation.

Findings showed the value of a comprehensive trusted resource for eczema advice and support such as the ECO intervention that is easily accessible at any time, particularly in the context of a pandemic. It was reassuring to know that the ECO intervention was available, even by those who did not have a need to use it over the course of the trial. Young people needed stress management or emotional support, which could be readily provided within online resources, although others may need in‐person support.

Findings relating to telephone consultations highlight the importance of providing information before, during and after about the process and effectiveness of remote assessment. This can include guidance for patients/carers about taking photos, what to expect around the technology and the consultation, and reassurance from healthcare professionals that they are confident in diagnosis and management of the condition, if appropriate, based on the verbal description and/or photographs provided by the patient. Future research may wish to explore the impact that face‐to‐face or video consultation might have on reassuring the patient and how this can be enhanced.

## CONFLICTS OF INTEREST

The authors have no conflicts of interest or financial disclosures to declare.

## Supporting information

Supplementary Material
