# Supplementary figures and images for: How has the COVID‐19 pandemic affected eczema self‐management and help seeking? A qualitative interview study with young people and parents/carers of children with eczema
Source: Skin Health Dis. 2021 Jun 23;1(4):e59. doi: 10.1002/ski2.59 (PMC8420339; doi:10.1002/ski2.59)

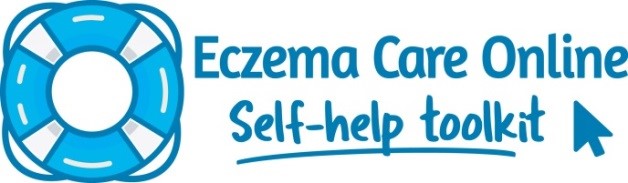

Supplement: Supplementary file 1 — Supplementary Material [file SKI2-1-e59-s001.jpg]
